# Supplementary material for: Structure of the complete, membrane-assembled COPII coat reveals a complex interaction network
Source: Nat Commun. 2021 Apr 1;12:2034. doi: 10.1038/s41467-021-22110-6 (PMC8016994; doi:10.1038/s41467-021-22110-6)
Supplement: Supplementary file 1 — Supplementary Information [file 41467_2021_22110_MOESM1_ESM.pdf]

Supplementary Information

Hutchings et al.

Structure of the complete, membrane-assembled COPII coat reveals a complex interaction network.

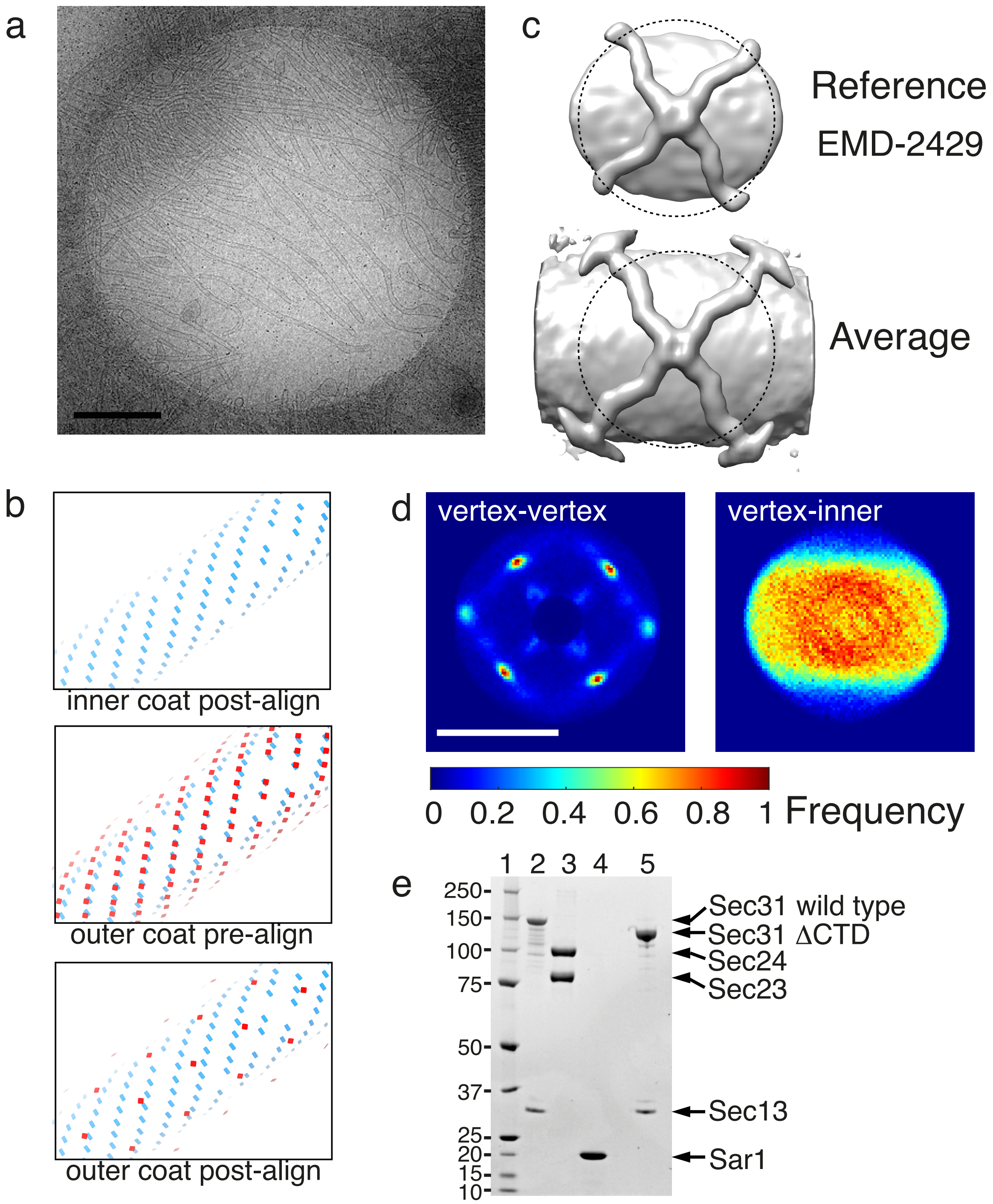

Supplementary Figure 1. Overview of subtomogram averaging.

a. A typical view of GUV budding on grids, representative of experiments repeated more than three times. Scale bar 1um.

b. Workflow for extraction of outer coat subtomograms. Top: aligned inner coat subunits arrange in a lattice. Middle: radially shifted coordinates from inner coat lattice points are used to extract outer coat particles. This oversamples the outer coat lattice. Bottom: alignments of outer coat particles with large shift in their xy plane but restricted shifts in the z direction leads to finding outer coat subunit accurately, as shown by the outer coat lattice pattern. This strategy was necessary to avoid the high-signal inner coat to bias the alignments.

c. Left panel: the starting reference is shown overlaid with the mask used. Right panel: the average obtained after one iteration clearly shows continuous density outside the mask, including neighbouring vertices and underlying smeared inner coat layer, indicative of genuine alignments.

d. Left panel: plot of the positions of neighbouring vertices to each aligned vertex shows the expected pattern. Right panel: plot of neighbouring inner coat subunits with respect to each vertex does not show any pattern, indicating the two coat layers are disordered with respect to each other. Scale bar 50 nm.

e. A Coomassie-blue stained gel of the proteins that were used in our cryo structural analyses. From lanes 1 to 5: 1. molecular marker, 2. Wild type Sec13-31, 3. Wild type Sec23-24, 4. Sar1, 5. Sec13-31ΔCTD. Molecular weight in KDa.

Source data are provided as a Source Data file.

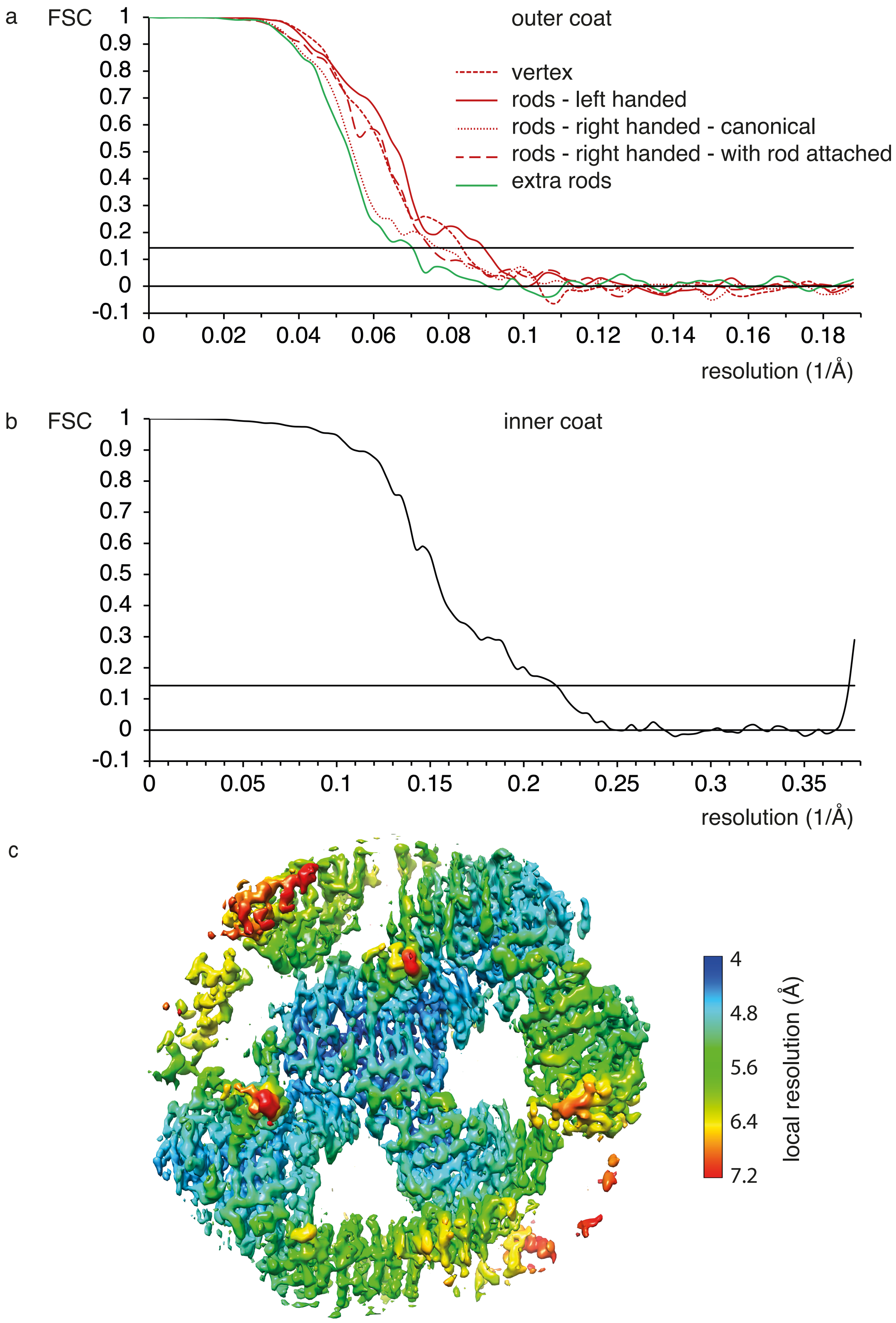

Supplementary Figure 2. Resolution of subtomogram averages.

a. Fourier Shell Correlation between independently processed half maps for the outer coat structures, colours as indicated in the legend. Line indicating FSC=0.143 is drawn.

b. Fourier Shell Correlation between independently processed half maps for the inner coat.

c. A map of the inner coat coloured according to its local resolution.

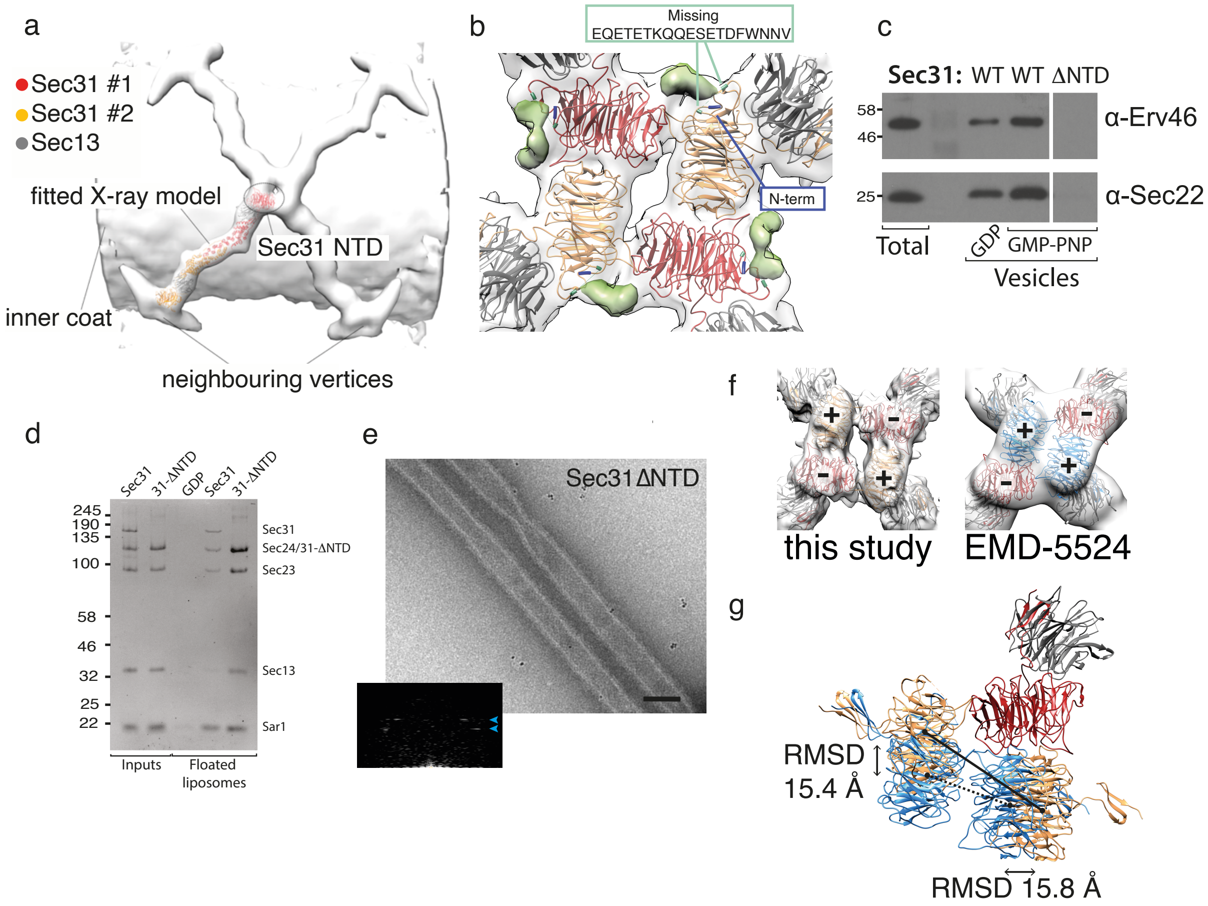

Supplementary Figure 3. Vertex interaction between Sec31 N-terminal β-propellers.

a. Fit of a Sec13-31 heterotetramer into a low-resolution map that includes neighbouring vertices. The X-ray-derived model unambiguously fits into the map, thereby identifying the identity of each domain, including the Sec31 N-terminal β-propeller as the protomers forming thee cage vertices.

b. A bottom view of the vertex reconstruction (outlined transparent white), with fitted models (Sec31 in dark red and orange, and Sec13 in grey). In the model, the N-terminal residues are coloured in blue, and residues that flank an acidic loop missing from the X-ray structures are coloured in green and indicated by a box with its aminoacidic sequence. The difference map between the subtomogram average and the model is shown in green, and is consistent with being occupied by the acidic loop. Sigma threshold for contouring was set at 2.2.

c. *In vitro* budding experiments using yeast microsomal membranes incubated with Sar1, Sec23/Sec24, Sec13 with the indicated mutant of Sec31, and the indicated nucleotides. Vesicle release from the donor membrane is measured by detecting incorporation of COPII cargo proteins, Sec22 and Erv46, into a slowly sedimenting vesicle fraction. When the NTD of Sec31 is missing, no budding is detected. Molecular weight markers in KDa. A representative result is shown from at least three replicates.

d. Flotation assays measure recruitment of COPII proteins to buoyant liposomes. Sec31-ΔNTD is recruited with similar efficiency to wild type Sec31, indicating that the budding and viability defects are not due to low recruitment levels. Molecular weight markers in KDa.

e. Negatively stained GUV budding reconstitutions performed with the full set of COPII proteins, but where Sec31 was deleted of its N-terminal β-propeller domain. These show tubulation and ordered inner coat lattice. Scale bar 100 nm. A detail of the power spectrum of a tube is shown in the inset, with the inner coat helical lattice layer lines clearly visible (blue arrows). This micrograph is representative of experiments repeated at least three times.

f. Comparison of the vertex reconstructed from coated membrane tubules (this study), with that of cages obtained in the absence of membranes [32]. The '-' protomers are in dark red, and the '+' protomers are shown in orange and blue respectively. Sigma threshold for contouring was set at 2.2.

g. The fitted models were superimposed on a '-' Sec31 protomer, and the relative positions of the neighbouring '+' protomers are overlapped, highlighting the differences between the two architectures.

Source data are provided as a Source Data file.

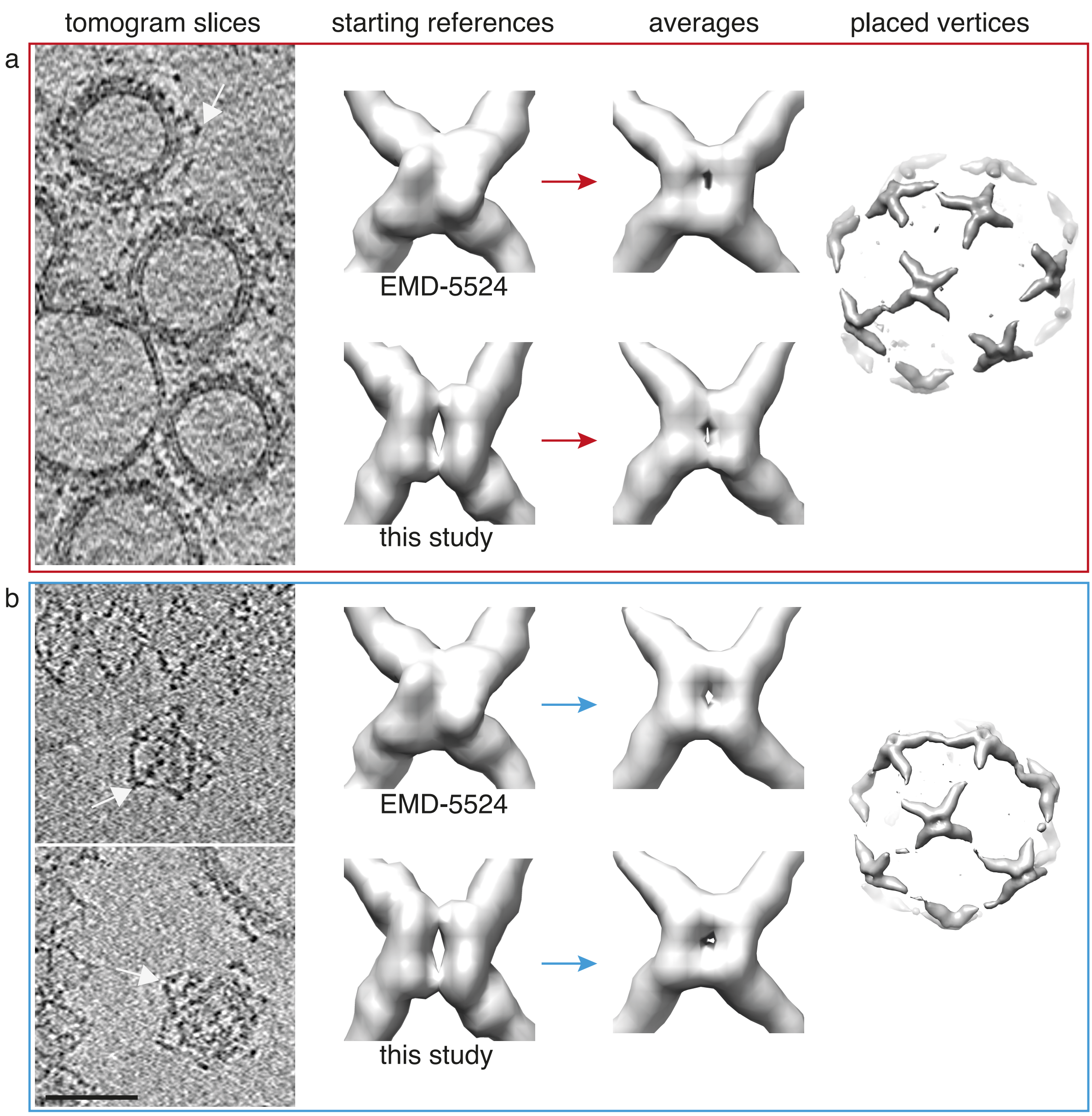

Supplementary Figure 4. Vertices on round vesicles and empty cages are hollow.

a. 79 vertices were manually picked from spherical vesicles found throughout the tomograms used for structural determination on tubules, and aligned against starting references originated either from a low-pass filtered version of the vertex obtained from tubules, or to the vertex of soluble cages obtained through in vitro assembly in the absence of membranes. In both cases, averages show a hollow architecture, more similar to the vertex arrangement we see on membrane tubules. Places vertex confirm the alignments are correct.

b. As in 'a', but 205 vertices were manually picked from membrane-less cages that were found in the cryo-tomograms as by-products of budding reactions. Scale bar 50 nm.

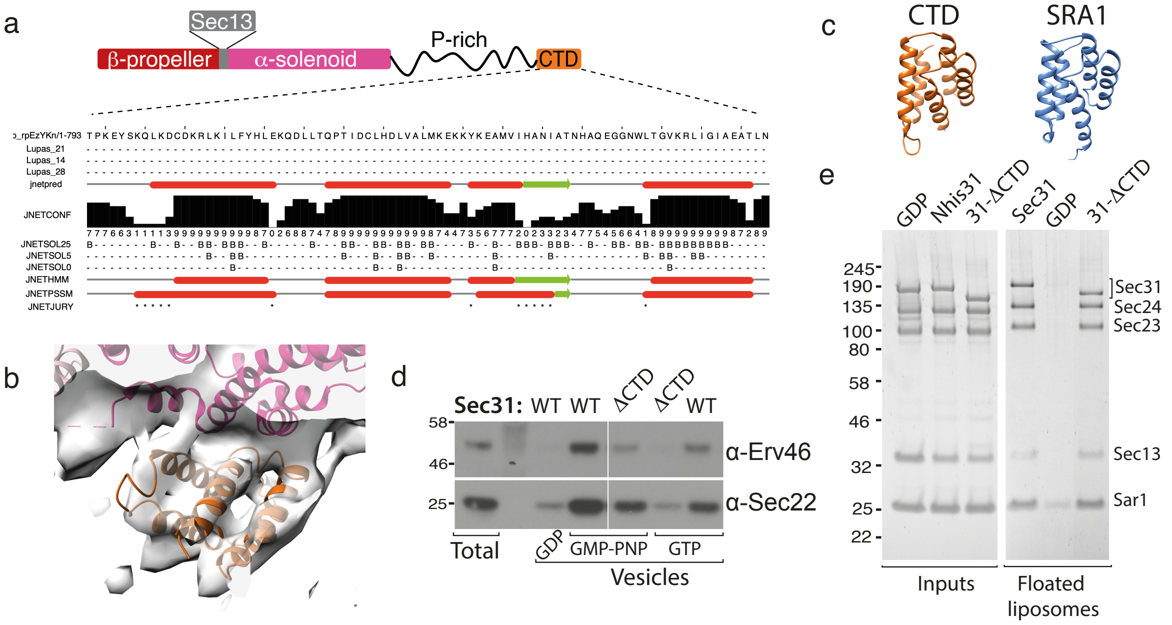

Supplementary Figure 5. Sec31 CTD characterisation.

a. Secondary structure predictions of Sec31 CTD indicate the presence of a folded domain, consisting mainly of α-helices.

b. A homology model for the Sec31 CTD was built based on SRA1, which was detected in sequence homology searches as the best match. The homology model was fitted to the appendage density and shows consistency of features. Sigma threshold for contouring was set at 2.2.

c. Side by side comparison of the homology model of the CTD with the SRA1 template used.

d. As in Supplementary Fig. 4c. When Sec31 CTD is missing, weak budding is detected with non-hydrolysable GTP analogues but not with GTP. Molecular weight markers in KDa. A representative result is shown from at least three replicates.

e. As in Supplementary Fig. 4c. Sec31-ΔCTD is efficiently recruited to membranes, indicating the functional defects are not a consequence of low recruitment levels. Molecular weight markers in KDa.

Source data are provided as a Source Data file.

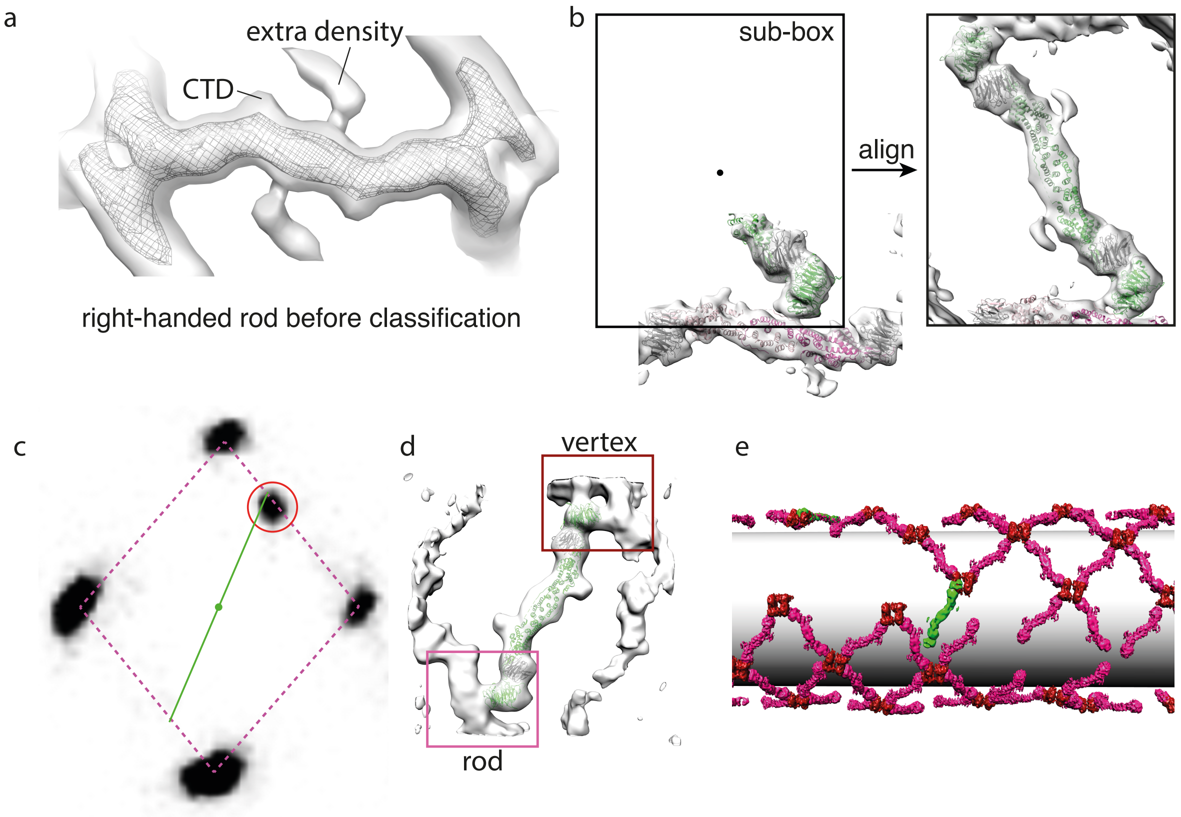

Supplementary Figure 6. Analysis of extra rods connecting between right-handed rods.

a. Right handed rods after the first round of alignments (bin x 8) showed prominent extra density.

b. After classification, a class emerged of right-handed rods which had a structured domain attached to the dimerization interface, resembling a tandem of β-propeller domains of Sec13-31 rods. Sub-boxing was performed by extracting particles at the predicted centre of the extra rods, and subtomogram averaging and subsequent alignments clearly converged into a Sec13-31 rod structure.

c. The plotted position of vertices neighbouring the extra rods. Given the extra rods position within the outer coat lattice (green line), vertices are predicted to position at the corners of a rhombus (dotted pink lines). An extra peak was detected in the neighbour plot, which corresponds to the tip of the extra rod (red circle).

d. Selecting and averaging extra rod particles based on masks on the neighbour plot clearly shows that some of these rods are connected to a right-handed rod on one side, and a vertex on the other.

e. Rods selected in 'd' (green) were placed in their original positions and orientation in a representative tomogram, together with the canonical rods and vertices (dark pink and red, respectively), showing that they bridge between patches of mismatched outer coat lattice.

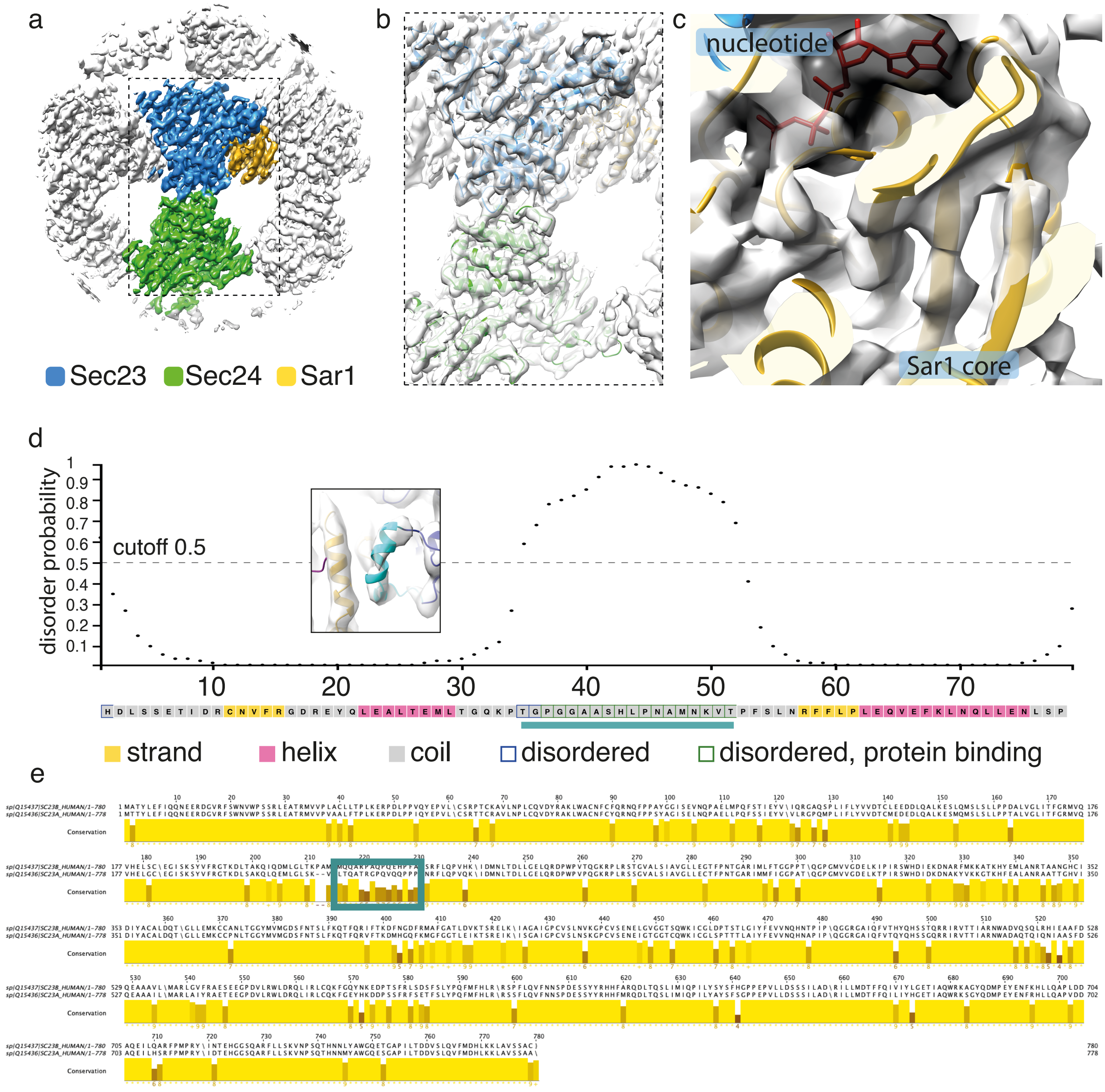

Supplementary Figure 7. Inner coat subtomogram averaging.

a. Overview of the sharpened map, with the central subunit coloured according to the model.

b. The X-ray-based model of Sec23, Sec24 and Sar1 was refined in the map, as detailed in the methods.

c. A close up view of the model in the core of Sar1, showing clear separation of β-strands and density for the bound nucleotide. Sigma threshold for contouring was set at 1 for panels 'a', 'b' and 'c'.

d. Disorder prediction of the Sec23 region around the L-loop (inset), as done with the PSIPRED server [60,61].

e. Alignment between human Sec23A and B paralogues shows very high conservation throughout the proteins, aside of a short region corresponding to the L-loop.

Supplementary Tables:

Table 1: List of primers

Primer name	Sequence (5' – 3')	Function
Sec31-fo	CTGTATTTTCAGGGC ATGGTCAAACTTGCTGAGTTTTCTC	In-Fusion with pFASTbacHTb-re (includes HIS and TEV)
Sec31-re	CTTGGTACCGCATGC *TTA*ATTCAAAGTCGCTTCAGCTATG	In-Fusion with pFASTbacHTb-fo. *TTA* is stop
Sec13-fo	CGGTCCGAAACCATG ATGGTCGTCATAGCTAATGCGC	In-Fusion with pFASTbacHTb-notag-re (no HIS and TEV).
Sec13-re	CTTGGTACCGCATGC *TCA*CTGATGAACTTCACCAGCG	In-Fusion with pFASTbacHTb-fo. *TCA* is stop.
Sec24-fo	CTGTATTTTCAGGGC ATGTCTCATCACAAGAAACGTGTTTAC	In-Fusion with pFASTbacHTb-re (includes HIS and TEV)
Sec24-re	CTTGGTACCGCATGC *TTA*TTTGCTAATTCTGGCTTTCATG	In-Fusion with pFASTbacHTb-fo. *TTA* is stop.
Sec23-fo	CGGTCCGAAACCATG ATGGACTTCGAGACTAATGAAGACATC	In-Fusion with pFASTbacHTb-notag-re (no HIS or TEV).
Sec23-re	CTTGGTACCGCATGC *CTA*TGCCTGACCAGAGACGG	In-Fusion with pFASTbacHTb-fo. *CTA* is stop.
pFASTbacHTb-re	GCCCTGAAAATACAGGTTTTCGGTC	Reverse primer to amplify pFASTBacHT-b up to HIS and TEV cleavage site.
pFASTbacHTb-notag-re	CATGGTTTCGGACCGAGATCCG	Reverse primer to amplify pFASTBacHT-b (no HIS and TEV).
pFASTbacHTb-fo	GCATGCGGTACCAAGCTTGTC	Forward primer to amplify pFASTBacHT-b.
Sar1-fo	CTTTATTTTCAGGGC ATGGCTGGTTGGGATATTTTTG	In-Fusion with petm11-re.
Sar1-re	ATCCGGTACCACTAG *TTA*AATATATTGAGATAACCATTGGAACG	In-Fusion with petm11-fo. *TTA* is stop.
Sar1-intr-fo	GGTTGGT TCAGAGATGTGTTGGCTTCCC	Amplify Sar1 insert before intron + 7 sticky bases after intron.

Sar1-intr-re	ATCTCTGA ACCAACCAAAAATATCCCAACC	Amplify Sar1 insert after intron + 8 sticky bases before intron.
petm11-fo	CTAGTGGTACCGGATCCGAATTC	Forward primer to amplify pETM-11.
petm11-re	GCCCTGAAAATAAAGATTCTCAGTAGTG	Reverse primer to amplify pETM-11.
sec31p_deltaCTerm_R	ACATTTATCTCAGCGGAGGTATTAACCTGAG	Reverse Primer to clone Sec31 ΔCTD in pFASTbacHTb
Sec31p_NTDd373_CHis_f	TCTCGGTCCGAAACCATGGCCCCAACTTGGTATGGGG	Forward Primer to clone Sec31 ΔNTD in pFASTbacHTb

Table 2: Data collection parameters

	WT COPII (1)	WT COPII (2)	ΔCTD
Grids	C-flat holey carbon coated gold grids (CF-4/1-4AU, Electron Microscopy Sciences)	C-flat holey carbon coated gold grids (CF-4/1-4AU, Electron Microscopy Sciences)	Lacey Carbon film, 200 Copper, (S166-3, Agar Scientific)
Cryo-specimen freezing	Vitrobot Mark IV	Vitrobot Mark IV	Leica EM GP
Electron microscope	Titan Krios, EMBL	Titan Krios, Birkbeck	Titan Krios, Birkbeck
Detector	K2 direct detector, Gatan	K2 direct detector, Gatan	K3 direct detector, Gatan
Datasets	1	1	1
Micrographs (used in processing)	137 tomograms	149 tomograms	47 tomograms
Voltage (keV)	300		
GIF energy filter slit width (eV)	20		
Electron exposure (e^{-}/Å^2)	~ 120	~ 150	~ 120
Sampling interval	1.33 Å/pixel	1.327 Å/pixel	0.69 Å/pixel (superresolution), 1.38 Å/pixel 2x2 binned.
Exposure time	0.25 sec frame / 0.75 sec total	0.1 sec frame / 1 sec total	0.08 sec frame / 0.4 sec total
Defocus range	-1.5 to -4.5 µm	-1.5 to -4.5 µm	-1.5 to -3.5 µm
Defocus determination	CTFFIND4		

Table 3: 3D STA reconstructions and model refinement

	Inner coat	Outer coat			
		Vertex	Left-handed rod	Right-handed rod	Right-handed rod with extra
Particle box size	196	128	128	128	128
Number of particles used	151,176	14,099	16455	7958	7195
Initial map generation	Dynamo	Dynamo	Dynamo	Dynamo	Dynamo
Map refinement	Dynamo	Dynamo	Dynamo	Dynamo	Dynamo
Resolution	4.6 Å	12 Å	11 Å	13 Å	15 Å

Table 4: refinement statistics for the inner coat

Model composition	
Protein residues	3413
No. Ligands	8
Protein atoms	26852
Ligand atoms	70
Model Resolution @ FSC 0.5 (Å)	4.6
Map CC (mask)	0.716
Average B-factor (all atoms) (Å^2)	158.4
RMS deviations – Bonds (Å)	0.01
RMS deviations – Angles (deg)	1.52
Molprobity Score	2.18
Clashscore	13.11
Ramachandran plot (%)	
Favoured	89.7
Allowed	10.1
Outlier	0.21
C-beta deviations (%)	0.00
Rotamer Outliers (%)	0.07
