# Supplementary material for: Structure of the complete, membrane-assembled COPII coat reveals a complex interaction network
Source: Nat Commun. 2021 Apr 1;12:2034. doi: 10.1038/s41467-021-22110-6 (PMC8016994; doi:10.1038/s41467-021-22110-6)
Supplement: Supplementary file 3 — Reporting Summary [file 41467_2021_22110_MOESM3_ESM.pdf]

nature research

Corresponding author(s): Giulia Zanetti

Last updated by author(s): Feb 5, 2021

Reporting Summary

Nature Research wishes to improve the reproducibility of the work that we publish. This form provides structure for consistency and transparency in reporting. For further information on Nature Research policies, see our Editorial Policies and the Editorial Policy Checklist.

Statistics

For all statistical analyses, confirm that the following items are present in the figure legend, table legend, main text, or Methods section.

n/a	Confirmed	
☐	☒	The exact sample size (*n*) for each experimental group/condition, given as a discrete number and unit of measurement
☒	☐	A statement on whether measurements were taken from distinct samples or whether the same sample was measured repeatedly
☒	☐	The statistical test(s) used AND whether they are one- or two-sided<br>*Only common tests should be described solely by name; describe more complex techniques in the Methods section.*
☒	☐	A description of all covariates tested
☒	☐	A description of any assumptions or corrections, such as tests of normality and adjustment for multiple comparisons
☒	☐	A full description of the statistical parameters including central tendency (e.g. means) or other basic estimates (e.g. regression coefficient) AND variation (e.g. standard deviation) or associated estimates of uncertainty (e.g. confidence intervals)
☒	☐	For null hypothesis testing, the test statistic (e.g. *F*, *t*, *r*) with confidence intervals, effect sizes, degrees of freedom and *P* value noted<br>*Give P values as exact values whenever suitable.*
☒	☐	For Bayesian analysis, information on the choice of priors and Markov chain Monte Carlo settings
☒	☐	For hierarchical and complex designs, identification of the appropriate level for tests and full reporting of outcomes
☒	☐	Estimates of effect sizes (e.g. Cohen's *d*, Pearson's *r*), indicating how they were calculated

Our web collection on statistics for biologists contains articles on many of the points above.

Software and code

Policy information about availability of computer code

Data collection	Serial EM (v3.8) was used for data collection on the Titan Krios 300kV electron microscope at the Birkbeck (London) and EMBL (Heidelberg). For tomography, the dose-symmetric tilt scheme was used as described in Hagen et al (2017). The scripts for this are also publicly available on the SerialEM repository.
Data analysis	Motioncor2 (v1.3) was used to for frame alignment. IMOD (v4.9.0) and Dynamo (v1.1.471) were used for the reconstruction of tomograms from collected tilt series. Matlab (v R2018a) and Dynamo were used in the processing of this data. Custom Matlab scripts were also used in parts of the processing pipeline. CTFFIND4 (v4.0.17) and NovaCTF (v1.0) were used for 3D-CTF estimation and correction. Data visualisation and analysis was done in UCSF Chimera (v1.13). RELION (v3.1) and (v1.18) were used for assessment of global and local resolution and density modification.

For manuscripts utilizing custom algorithms or software that are central to the research but not yet described in published literature, software must be made available to editors and reviewers. We strongly encourage code deposition in a community repository (e.g. GitHub). See the Nature Research guidelines for submitting code & software for further information.

Data

Policy information about availability of data

All manuscripts must include a data availability statement. This statement should provide the following information, where applicable:

- Accession codes, unique identifiers, or web links for publicly available datasets
- A list of figures that have associated raw data
- A description of any restrictions on data availability

We have deposited the EM maps and models to the Electron Microscopy and Protein Data Banks with accession codes: EMD-11193,11194,11197,11198,11199,11264 and PDB 6ZG5,6ZG6,6ZGA,6ZL0 .

Databases and servers used: PDB, CATH, Uniprot, HHpred, proSA.

Field-specific reporting

Please select the one below that is the best fit for your research. If you are not sure, read the appropriate sections before making your selection.

☒ Life sciences ☐ Behavioural & social sciences ☐ Ecological, evolutionary & environmental sciences

For a reference copy of the document with all sections, see nature.com/documents/nr-reporting-summary-flat.pdf

Life sciences study design

All studies must disclose on these points even when the disclosure is negative.

Sample size	Total numbers of contributing particles for subtomogram averaging are stated in Table 2. The sample size was determined by the availability of data collection time.
Data exclusions	Tilt series were discarded on occasions where accurate tomogram reconstructions could not be achieved. For example, too few fiducials in a field of view, or excessive stage drift during acquisition.
Replication	For subtomogram averaging, all datasets were divided into two halves for independent processing. Map resolution was determined by half-map Fourier Shell Correlation (FSC), consistent with gold-standard methods.<br>All yeast viability tests, liposome binding, and microsome budding assays were repeated three times with consistent results, with representative results shown.
Randomization	Particles falling into the two halves for independent processing (see above) were selected randomly.
Blinding	Blinding was not relevant to this study as we manually selected areas of the imaged specimen for cryo-tomography data collection. For this study, we were interested in the tubular morphologies formed by COPII (see figure S1). As described above for the Replication and Randomization fields, the gold-standard approach to cryo-EM data processing was followed.<br>As described in the methods for subtomogram averaging, the alignments always used a reference that was low-pass filtered to a resolution lower than that judged by FSC between the two half datasets, with a 0.5 cut-off. This means that emergence of features with resolution better than that of the filter occurs without bias from the reference.<br>For other experiments, blinding was not relevant as groups were not allocated and all results were considered.

Reporting for specific materials, systems and methods

We require information from authors about some types of materials, experimental systems and methods used in many studies. Here, indicate whether each material, system or method listed is relevant to your study. If you are not sure if a list item applies to your research, read the appropriate section before selecting a response.

Materials & experimental systems

n/a	Involved in the study	
☐	☒	Antibodies
☐	☒	Eukaryotic cell lines
☒	☐	Palaeontology and archaeology
☒	☐	Animals and other organisms
☒	☐	Human research participants
☒	☐	Clinical data
☒	☐	Dual use research of concern

Methods

n/a	Involved in the study	
☒	☐	ChIP-seq
☒	☐	Flow cytometry
☒	☐	MRI-based neuroimaging

Antibodies

Antibodies used	Homemade rabbit polyclonal anti-Sec22 (from Elizabeth Miller) and anti-Erv46 (courtesy of Charles Barlowe) antibodies were used for blots.
Validation	Both of these antibodies were used in Pagant et al 2007, Mol. Biol. Cell.

Eukaryotic cell lines

Policy information about cell lines

Cell line source(s)	Sf9 insect cells for protein expression are from Invitrogen. LMY1249 and VSY015 yeast strains were used for viability tests.

Authentication	No authentication done.
Mycoplasma contamination	Cell lines were not tested for Mycoplasma contamination.
Commonly misidentified lines (See ICLAC register)	No misidentified cell lines were used in this study
